# Interstial granulomatous dermatitis as initial presentation of juvenile mixed connective disease and its response to tacrolimus

**DOI:** 10.1186/1546-0096-10-S1-A100

**Published:** 2012-07-13

**Authors:** Lawrence Jung, Igor Shendrik

**Affiliations:** 1 Children's National Medical Center, Washington, DC, USA; 2 St. John's Medical Center, Tulsa, OK, USA

## Purpose

Interstitial granulomatous dermatitis (IGD) has been reported in association with autoimmune disorders in adults. These associated conditions include rheumatoid arthritis, seronegative arthritis, SLE, autoimmune thyroiditis and others. It has also been described in drug-reaction pulmonary coccidioidomycosis, Lyme’s disease and pulmonary silicosis. IGD has rarely been described in children.

## Methods

A thirteen-year old white male presented with anorexia, fever (104^o^F) and joint pains involving his ankles, knees, elbows and shoulders. He developed multiple erythematous, plaques on chest and back. The patient was initially treated with prednisone, and the rash and joint symptoms resolved but recurred repeatedly. The rash was erythematous, slightly raised, tender for about 3 days, became ecchymotic and then gradually faded away in 7-10 days. The majority of the lesions were distributed on trunk and axilla. The lesions were non-pruritic, papular or annular in character and tended to migrate from one location to another.

**Figure 1:**
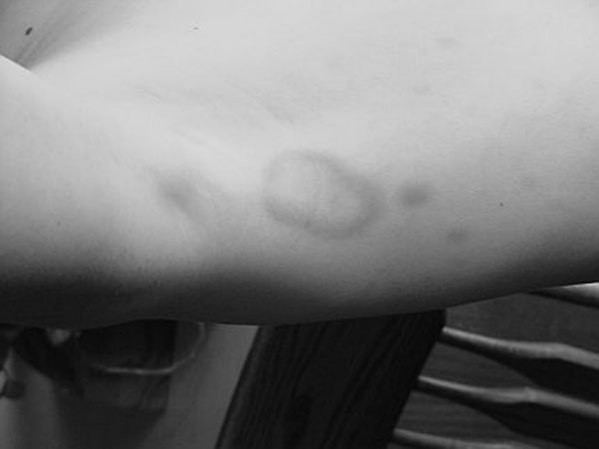


Microscopically, the lesion showed diffuse infiltration of the dermis and upper subcutaneous fat by mononuclear cells. The infiltrate had a perivascular and diffuse pattern and it splayed the pre-existing collagen fibers and extended to superficial fat, where it assumed a lobular pattern. Churg-Strauss granulomas were present, comprised of degenerated collagen foci with surrounding accumulation of histiocytes. Mild mucin accumulation was seen. The pattern was felt to be compatible with IGD.

**Figure 2:**
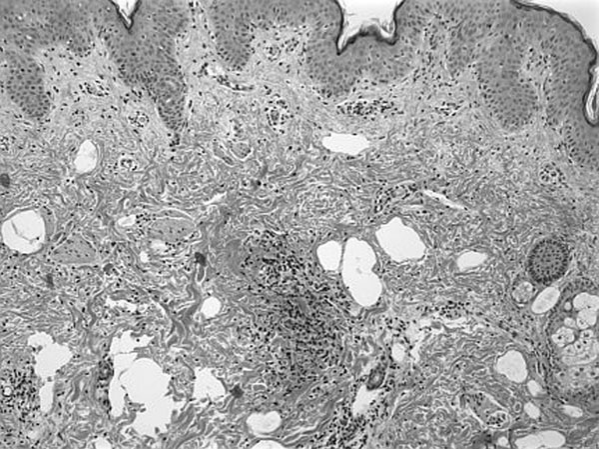


Immune work-up revealed the following: FANA- 1:1280; anti-nRNP 1:512; with negative anti-dsDNA, anti-Sm., anti-Scl70, anti-SSA/B, and anti-centromere antibodies. ANCA was negative. CPK was elevated at height of muscle weakness at 840 IU (NR <200) while Aldolase was 24.0 u/l (NR < 8.3 u/l). Studies for infectious etiology were negative.

## Results

Although the patient’s symptoms responded to oral prednisone, his symptoms returned as steroid therapy was withheld. Immunosuppressive agents such as methotrexate and cyclosporine were ineffective in controlling the disease but responsive to tacrolimus was dramatic and sustained.

## Conclusion

IGD is an uncommon dermatologic manifestation of pediatric rheumatic diseases. This case is a first description of its occurrence in childhood mixed connective disease and pediatric rheumatology community should be aware of its presentation. The response of IGD to tacrolimus in this patient may reflect the underlying immunopathogenesis of IGD. Further, tacrolimus should be considered in recalcitrant cases of IGD.

## Disclosure

Lawrence Jung: None; Igor Shendrik: None.

